# Dissociable effects of the apolipoprotein-E (APOE) gene on short- and long-term memories

**DOI:** 10.1016/j.neurobiolaging.2018.09.017

**Published:** 2019-01

**Authors:** Nahid Zokaei, Giedrė Čepukaitytė, Alexander G. Board, Clare E. Mackay, Masud Husain, Anna Christina Nobre

**Affiliations:** a Department of Psychiatry, Oxford Centre for Human Brain Activity, Wellcome Centre for Integrative Neuroimaging, University of Oxford, Oxford, UK; b Department of Experimental Psychology, University of Oxford, Oxford, UK; c Department of Psychiatry, University of Oxford, Oxford, UK; d Nuffield Department of Clinical Neurosciences, University of Oxford, Oxford, UK

**Keywords:** Short-term memory, Long-term memory, Alzheimer's disease, Apolipoprotein-E

## Abstract

Short- and long-term memory performance as a function of apolipoprotein-E (APOE) genotype was examined in older, healthy individuals using sensitive and comparable tasks to provide a more detailed description of influences of the ε4 allele (highest genetic risk factor for Alzheimer's disease) on memory. Older heterozygous and homozygous ε4 carriers and noncarriers performed 2 tasks of memory. Both tasks allowed us to measure memory for item identity and locations, using a sensitive, continuous measure of report. Long-term memory for object locations was impaired in ε4/ε4 carriers, whereas, paradoxically, this group demonstrated superior short-term memory for locations. The dissociable effects of the gene on short- and long-term memory suggest that the effect of genotype on these two types of memories, and their neural underpinnings, might not be co-extensive. Whereas the long-term memory impairment might be linked to preclinical Alzheimer's disease, the short-term memory advantage may reflect an independent, phenotypical effect of this allele on cognition.

## 1 Introduction

The prevalence of Alzheimer's disease (AD) increases dramatically with age, with ∼13% of individuals over the age of 65 years and 45% above 85 years being diagnosed with AD (Alzheimer's Association, 2016, Liu et al., 2013). Prodromal AD—the period between the onset of the neurodegeneration and diagnosis—is likely to be the optimal time to introduce any disease-modifying treatments. Prodromal AD is often associated with several cognitive deficits, which might provide a means for early detection. Searching for such impairments on a population-wide basis is challenging; an alternative method might be to examine at-risk cohorts instead.

One such group is carriers of the apolipoprotein-E (APOE) ε4 gene allele. APOE ε4 confers the highest known genetic risk for developing sporadic AD in older age, with 30%–60% of those diagnosed with AD carrying 1 or 2 copies of the APOE ε4 allele (Myers et al., 1996, Sando et al., 2008, Saunders et al., 1993, Winblad et al., 2016). The high risk of conversion makes individuals with ε4 allele ideal candidates for investigating cognitive impairments that may point to early signs of AD (Bondi et al., 2008, O’Donoghue et al., 2018, Weintraub et al., 2012).

Long-term memory (LTM) deficits, which are often associated with AD, are also observed in ε4 carriers compared with noncarriers (Small et al., 2004, Wisdom et al., 2011, Wolk et al., 2010). In patients with AD, ε4 carriers had a larger deficit in delayed recall tasks, with memory delays of few minutes, compared with noncarriers (Wolk et al., 2010). Studies using neuropsychological measures of verbal and nonverbal delayed recall have reported impaired memory in healthy young (Nao et al., 2017), middle-aged (Caselli et al., 2001, Flory et al., 2000), and older (Caselli et al., 2009, Caselli et al., 2011, Deary et al., 2002, Levy et al., 2004) ε4 carriers compared with noncarriers. These findings have been taken to be indicative of the prodromal stages of AD in these at-risk groups.

Paradoxically, compared to LTM performance, in patients with AD, ε4 carriers performed significantly better in immediate memory recall tasks, with memory delays of a few seconds in length (Wolk et al., 2010). In healthy participants, although the evidence is scarce, a few studies have reported superior cognitive performance in ε4 carriers, specifically in tasks of short-term memory (STM) (Carrión-Baralt et al., 2009, Greenwood et al., 2005, Greenwood et al., 2014, Mondadori et al., 2007, Wolk et al., 2010, Zokaei et al., 2017). Such evidence has been taken for a phenotypic effect of APOE on cognition independent of AD pathology, which might also account for the survival of this genotype through a mechanism of antagonistic pleiotropy (Williams, 1957).

Cognitive differences in ε4 carriers are accompanied by differences in brain structure and function. In patients with AD, quantitative neuroimaging studies have reported greater medial temporal lobe (MTL) atrophy in ε4 carriers compared with noncarriers (Agosta et al., 2009, Geroldi et al., 1999, Hashimoto et al., 2001, Lehtovirta et al., 1995, Wolk et al., 2010). Middle-aged healthy ε4 carriers displayed reduced functional magnetic resonance imaging activation in the MTL and hippocampal regions during an LTM task, accompanied by equivalent behavioral performance to noncarriers (Trivedi et al., 2006). On the other hand, in patients with AD, ε4 carriers had less frontal lobe atrophy than noncarriers (Hashimoto et al., 2001, Wolk et al., 2010). In the healthy population, ε4 carriers exhibited increased activation in frontal regions during tasks of attention and STM (Scheller et al., 2017, Trachtenberg et al., 2012, Wishart et al., 2006).

These seemingly contradictory behavioral and neural differences in ε4 carriers are difficult to reconcile within just the prodromal or phenotypical frameworks. The 2 accounts, however, are not necessarily mutually exclusive. Whereas impairments in LTM might be a manifestation of prodromal stages of AD, the superior STM performance could instead reflect either phenotypical effects of the APOE gene or compensatory mechanisms that arise due to AD--related or non-AD-related pathology linked to APOE (Bondi et al., 2008, Han et al., 2007, Scheller et al., 2017). Although the association between the APOE ε4 and occurrence of AD is strong (Genin et al., 2011, Winblad et al., 2016), the APOE gene is also implicated in many complex processes in the central nervous system, for example, neurogenesis and plasticity, (Mahley and Rall, 2000) and linked to other neurological disorders, such as neurovascular dysfunction (Greenberg et al., 1996, Premkumar et al., 1996).

Therefore, 1 important gap in the literature is to bring together these 2 lines of research and provide an in-depth profile of memory in ε4 carriers that encompasses sensitive measures of cognitive functions classically associated with prodromal as well as phenotypic effects of the APOE gene. This will enable a more detailed description of influences of the ε4 alleles of the APOE gene on cognition and in turn aid the identification and development of a selective cognitive biomarker for AD. In the present study, we examined the effect of the APOE gene on STM and LTM in tasks that examined similar aspects of memory for object locations in healthy older carriers and noncarriers of the ε4 allele. Importantly, in the context of the current research, STM refers to conditions with memory delays of 1 or 8 seconds, whereas LTM delays lasted approximately 20 minutes. Furthermore, the older age group was examined because the phenotypic influences of APOE on cognition might be more pronounced in later life (McClearn et al., 1997, McGue and Christensen, 2002).

The direct effect of APOE on memory might be subtle and not always reliably detectable with standard neuropsychological measures that are commonly used to identify cognitive impairments in disease rather than variations in health. Ideally, the tasks should thus be sensitive to subtle differences in performance. Therefore, in our study, we examined STM and LTM using sensitive cognitive tasks that have previously been successful in detecting subtle changes in performance in healthy aging, neurodegenerative disorders, and in at-risk populations (Rolinski et al., 2016, Salvato et al., 2016b, Zokaei et al., 2017). Both tasks provided comparable measures of memory, specifically by examining the resolution with which locations were retained, using continuous, analogue measures of location memory. These contrast with standard measures that use discrete, binary (correct/incorrect) responses. Thus, this study has the potential to allow us to identify whether any differences in performance in ε4 carriers is attributable to maintenance of selective types of information across timescales (i.e., remembering a location irrespective of duration), types of processes (STM vs. LTM), or both.

## 2 Materials and methods

### 2.1 Participants

Sixty-six individuals participated in this study. They were invited according to their APOE allelic variants, through the NIHR BioResource (for APOE genotyping methods please refer to the NIHR BioResource website: https://bioresource.nihr.ac.uk/). For the present study, participants with *APOE ε3/ε3, ε3/ε4,* and *ε4/ε4* genotypes were invited to participate, 22 (11 males and 11 females) per group (see Table 1 for demographics). Neither the experimenter nor the participants were aware of the genetic status at the time of testing (double-blind protocol).

**Table 1:** Demographic characteristics of the final sample (11 male and females per group)

APOE genotypes	Age, mean (SD)	Handedness (R/L)	Years of education, mean (SD)	ACE-attention, mean (SD)	ACE-memory, mean (SD)	ACE-fluency, mean (SD)	ACE-language, mean (SD)	ACE-visuospatial, mean (SD)
ε3/ε3	69.7 (4.8)	19/3	16.5 (3)	17.4 (1.2)	25 (1.5)	12.7 (1.3)	26 (0.3)	15.5 (0.6)
ε4/ε3	68.7 (4.6)	20/2	17 (4)	17.4 (0.8)	24.6 (1.9)	12.8 (1.3)	25.6 (0.6)	14.5 (0.9)
ε4/ε4	68.1 (5)	21/1	16.2 (3)	17.2 (0.8)	23.5 (3.2)	11.7 (1.7)	25.5 (0.6)	15.5 (0.6)
Significance (*p*)	0.5	0.6	0.2	0.4	0.12	0.062	0.1	0.6

Key: ACE, Addenbrooke's Cognitive Examination.

All participants had normal or corrected-to-normal visual acuity and normal color vision. The study was approved by University of Oxford Ethics Committee. The Addenbrooke's Cognitive Examination III (ACE-III) test was administered as a general cognitive screening test to all participants (means scores in Table 1). None of the participants exhibited significant cognitive impairment using a cutoff of 88/100.

### 2.2 Computer-based tasks

Both STM and LTM tasks were presented on a touch screen (Inspiron All-in-One 2320; DELL) with a 1920 × 1080 pixel resolution (corresponding to 62°×35° of visual angle) at a viewing distance of approximately 62 cm.

#### 2.2.1 STM task

**Fig. 1:**
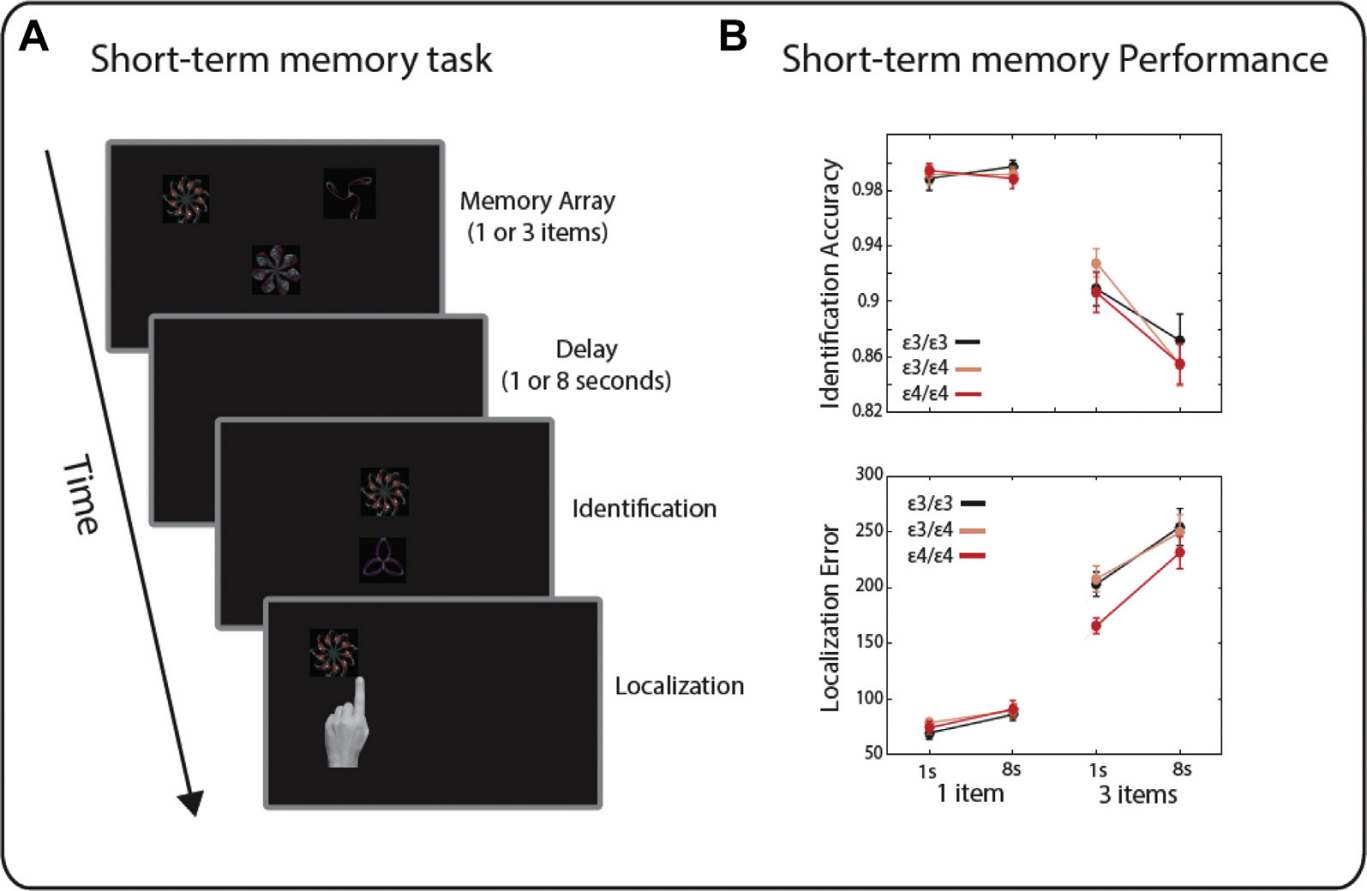
Short-term memory task performance. (A) A schematic of the short-term memory task. (B) Short-term memory task performance identification accuracy did not differ in individuals with differences in APOE alleles (upper). Localization performance (lower) demonstrated that ε4/ε4 carriers performed significantly more accurate in trials with 3 items than ε3/ε4 and ε3/ε3 carriers. Error bars correspond to standard error.

The STM task was identical to the one previously used (Zokaei et al., 2017, Zokaei et al., 2018) (Fig. 1A). Stimuli were randomly selected from a pool of 60 colored fractals, with maximal width and height of 120 pixels (4°of visual angle). Fractals were presented 2/3 times per block. The location of each fractal was random, but with a minimum distance of 9°of visual angle between fractals, a minimum distance of 3.9° from monitor edge, and a minimum distance of 6.5° from the center.

In each trial, participants were presented with 1 or 3 fractals (the memory array) for 1 or 3 seconds, respectively. The memory array was followed by either 1 or 8 seconds of blank screen. At recall, participants were first presented with 2 fractals on the vertical meridian at screen center. One of the items had been presented in the preceding memory array while the other was a foil, that is, a fractal that did not appear in the memory array. Participants were required to select the fractal that appeared in the memory array by tapping on the fractal (*identification accuracy*) and then drag it to its remembered location (*localization memory*). They confirmed their response with a key press.

Participants performed 2 blocks of 50 trials each (16 trials with 1 fractal in the memory array and 34 trials with 3). Half of the trials were followed by a delay of 1 second and half by 8 seconds. Before starting the task, participants were acquainted with the experimental apparatus and conditions by completing 10 practice trials.

#### 2.2.2 LTM task

##### 2.2.2.1 Stimuli

The LTM task was a modified version of a previously used paradigm (Salvato et al., 2016a, Salvato et al., 2016b), which provides measures of object identification accuracy and localization error, similar to the STM task.

The LTM array included 96 randomly selected digital colored photographs (1000 × 750 pixels) of complex indoor and outdoor scenes (from a set of 150). An additional fixed set of 12 scenes was used for practice trials. The scenes were overlaid with 1 or 2 colored images of objects (120 objects overall, from a pool of 415 objects). Object images were resized to fit within a 50 × 50 pixels transparent box (3.4° × 4.5° of visual angle). A further 48 novel objects were selected to be presented as foils during the explicit memory recall phase.

Locations of objects within the scene were pseudorandomized and were not semantically related to the scenes. Objects were placed at least 50 pixels away from both the center and the edges of the scene. Furthermore, in scenes with 2 objects, there was a minimum distance of 200 pixels between the two objects. Objects were placed equiprobably in all 4 quadrants of the scene, and similarly, the second object (in trials with 2 objects) was placed in each quadrant with equal probability.

##### 2.2.2.2 Task and procedure

**Fig. 2:**
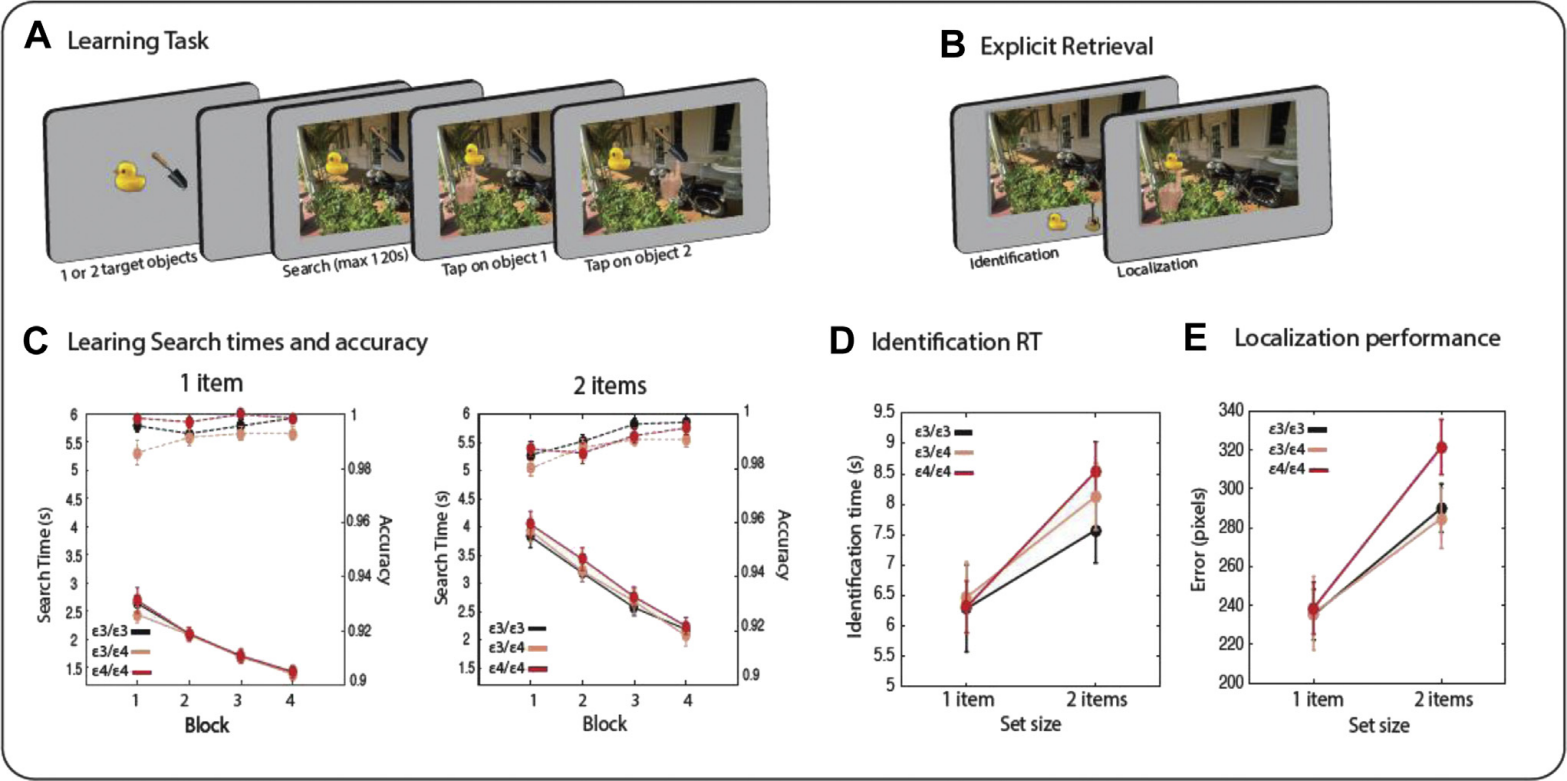
Long-term memory task and performance. A schematic of the learning (A) and explicit retrieval (following a 20-minute memory delay) (B) phase of the task. (C) Search times (solid lines) and accuracy (dashed lines) across the 4 blocks of the learning phase for trials with 1 object (on the left) and 2 objects (on the right) for individuals with different APOE alleles. (D) Identification response times demonstrated that ε4 carriers were slower than noncarriers. (E) ε4/ε4 carriers were impaired in localization performance in trials with 2 objects, that is, for larger set sizes. Error bars correspond to standard error.

Learning: A schematic of the learning phase of the LTM task is presented in Fig. 2A. At the beginning of each trial, 1 or 2 objects were presented centrally for 4 seconds. This was followed by a blank screen (250 ms) before the presentation of a scene containing the objects. Participants were asked to find the objects in the scene and press the space bar as soon as the objects were found. They then had to tap on the objects in scene to confirm object locations. The search period timed out after a maximum of 120 seconds. There was a 1-second fixation period between trials.

Participants completed 4 blocks, each consisting of all 96 object-scene combinations (32 trials with 1 object and 64 trials with 2 objects). The 96 object-scene combinations were picked at random for each participant and were presented in each block in a randomized order. Each block took ∼12–15 minutes to complete, and participants were encouraged to take short breaks between the blocks. Before starting the learning task, participants were acquainted with experimental apparatus and procedures by completing practice trials.

Explicit retrieval: Once the learning phase was completed, participants had a 20-minute break. During this period, they completed the ACE assessment and relaxed for the remaining time. The break was followed by a surprise memory test examining their explicit memory for object identities and their locations associated with each scene. In each trial, participants were presented with 1 scene alongside 2 objects (Fig. 2B). One of the objects was previously associated with the scene (in the learning phase) and the other one was a foil. Participants were asked first to pick the object associated with that scene (identification accuracy) and then to drag it precisely to its remembered location (location memory). They confirmed their response with a key press.

## 3 Results

### 3.1 Demographics

The groups did not differ significantly in age [F(2,63)= 0.8, *p* = 0.5], years of education [F(2,63)= 1.5, *p* = 0.2], handedness [χ^2^(2,*N* = 66) = 1.1; *p* = 0.6), family history of dementia (all *p* > 0.05), depression [F(2,63) = 0.2, *p* = 0.7], or apathy [F(2,63) = 0.3, *p* = 0.8]. ACE-III scores, for each group, are given in Table 1. There was no effect of group on the attention [F(2,63) = 0.87, *p* = 0.4], memory [F(2,63) = 2.2, *p* = 0.12], fluency [F(2,63) = 2.9, *p* = 0.062], language [F(2,63) = 2.08, *p* = 0.1], and visuospatial [F(2,63) = 0.5, *p* = 0.6] domains of the ACE examination.

Differences between male and female participants were examined by using gender as a between-subject factor. There was no main effect of gender or any interaction between gender, APOE, and any of the experimental factors reported below. We therefore excluded gender from further analysis.

### 3.2 Short-term memory

We first analyzed the accuracy and response time for identifying the correct fractal from the STM array. Separate repeated-measures ANOVAs were used, with set size and delay as within-subject factors and the APOE group as a between-subject factor. For identification accuracies, there was no significant main effect of group [F(2,63) = 0.1, *p* = 0.9] or any interaction between group and set size [F(2,63) = 0.2, *p* = 0.8], group and delay [F(2,63) = 1.67, *p* = 0.2], or three-way interaction between all factors [F(2,63) = 1.67, *p* = 0.2]. There was a significant main effect of set size [F(2,63) = 0.5, *p* = 0.6, η^2^_p_ = 0.76] and delay [F(1,63) = 21, *p* < 0.001, η^2^_p_ = 0.25] and a significant interaction between the two factors [F(1,63) = 22, *p* < 0.001, η^2^_p_ = 0.26, Fig. 1B].

For identification times, there was no effect of group [F(2, 63) = 2.3, *p* = 0.11], of interaction between group and set size [F(2, 63) = 0.6, *p* = 0.55], group and delay [F(2, 63) = 1.07, *p* = 0.35], or a three-way interaction between the factors [F(2, 63) = 0.23, *p* = 0.8]. There was, however, a significant main effect of set size [F(1, 63) = 1756, *p* < 0.001, η^2^_p_ = 0.96], delay [F(1, 63) = 134, *p* < 0.001, η^2^_p_ = 0.7], and an interaction between the two factors [F(1, 63) = 54, *p* < 0.001, η^2^_p_ = 0.45]. Trials in which the correct fractal was not identified were excluded from the subsequent analysis.

We next examined localization memory, by measuring the distance between reported and actual location of the target item. There was a significant interaction between set size and group [F(2, 63) = 5, *p* = 0.009, η^2^_p_ = 0.14] and a significant three-way interaction between group, set size, and delay [F(2, 63) = 3.2, *p* = 0.048, η^2^_p_ = 0.09]. There was no significant main effect of group [F(2,63) = 2.09, *p* = 0.13]. There was, however, a significant main effect of set size [F(1,63) = 564, *p* < 0.001, η^2^_p_ = 0.9], delay [F(1,63) = 60, *p* < 0.001, η^2^_p_ = 0.49], and a significant interaction between these two factors [F(1, 63) = 23, *p* < 0.001, η^2^_p_ = 0.27, Fig. 1B].

We followed up the significant three-way interaction with 2 further two-way ANOVAs per each memory set size. For set size 1, there was no significant interaction between delay and group [F(2, 63) = 1.08, *p* = 0.35], or a significant main effect of group [F(1, 63) = 0.84, *p* = 0.44]. There was, however, a significant effect of delay [F(2, 63) = 33, *p* < 0.001]. For set size 3, there was a significant main effect of group [F(2,63) = 3.7, *p* = 0.03, η^2^_p_ = 0.1], delay [F(1, 63) = 44, *p* < 0.001, η^2^_p_ = 0.4] and a significant interaction between group and delay [F(2, 63) = 3.25, *p* = 0.045, η^2^_p_ = 0.09].

Post hoc analyses were performed to examine the significant interaction between delay and group for trials with 3 items in memory. In trials with shorter delays, there was a significant main effect of group on localization performance [F(2, 63) = 8.7, *p* < 0.001, η^2^_p_ = 0.2]. Post hoc tests using Bonferroni correction revealed that *ε4/ε4* carriers performed significantly better than *ε3/ε4* carriers (*p* = 0.033) and *ε3/ε3* carriers (*p* < 0.001). There was no effect of group for trials with longer delay or a significant difference between *ε3/ε3 and ε3/ε4* carriers*.*

### 3.3 Long-term memory

#### 3.3.1 Learning

Search accuracy and response times (i.e., time it took to press the space bar indicating that target object/s were found) were used to examine object-scene learning across blocks. Two separate mixed ANOVAs were performed with accuracy and response times as the dependent variables, APOE status as a between-subject factor, and block and set size condition as within-subject factors.

For search accuracies (Fig. 2C dashed lines), there was no significant interaction between group and set size [*F*(2,63) = 1.04, *p* = 0.4] or group and block [*F*(6,189) = 1.5, *p* = 0.19] as well as no significant main effect of group [*F*(2,63) = 1.9, *p* = 0.8]. However, there was a significant main effect of set size [*F*(1, 63) = 9.6, *p* = 0.003, η^2^_p_ = 0.13], and block [*F*(3, 189) = 13.5, *p* < 0.001, η^2^_p_ = 0.17], as well as a significant interaction between set size and block [*F*(3, 189) = 4.5, *p* = 0.004, η^2^_p_ =0.07].

Trials in which the objects were not found were excluded from the analysis of response times. Furthermore, response times shorter than 100 ms or longer than 2.5 standard deviations from the mean were also excluded (∼1% of trials). Participants had to tap on the objects once they had pressed the space bar indicating that they have found the objects. Therefore, trials with tap-times >15 seconds were also excluded (less than 1% of trials). These would have been trials in which participants pressed the bar before having found the objects.

For response times (Fig. 2C solid lines), there was no significant main effect of group [*F*(2,63) = 1.8, *p* = 0.8] or interaction between group and set size [*F*(2,63) = 0.6, *p* = 0.6] or group and block [*F*(6,189) = 0.3, *p* = 0.9]. There was, however, a significant main effect of set size [F(1, 63) = 266, *p* < 0.001, η^2^_p_ = 0.8], and block [F(3, 189) = 217, *p* < 0.001, η^2^_p_ = 0.7], as well as a significant interaction between set size and block [F(3, 189) = 20, *p* < 0.001, η^2^_p_ = 0.25], highlighting the difference in search slopes between the 2 set size conditions.

#### 3.3.2 Explicit retrieval

We first examined object identification accuracy during retrieval. Separate mixed ANOVAs with identification accuracy and response times for identifying the correct object as dependent variables tested the effects of APOE status as a between-subject factor and set size as a within-subject factor. There was no effect of group [F(1,63) = 0.25, *p* = 0.78] and no interaction between set size and group [F(2,63) = 0.66, *p* = 0.5] for identification accuracy. There was, however, a significant main effect of set size [F(1,63) = 30.5, *p* < 0.001, η^2^_p_ = 0.3], with lower identification accuracy for trials with 2 items than trials with a single item.

For identification times, however, there was a significant interaction between set size and group [F(2,62) = 4.3, *p* = 0.018, η^2^_p_ = 0.12] and a significant main effect of set size [F(1,63) = 113, *p* < 0.001, η^2^_p_ = 0.6], with no significant main effect of group [F(2, 62) = 0.5, *p* = 0.6]. Thus, identification times were longer for trials with 2 items, and the magnitude of increase in response times were influenced by the APOE group of participants (Fig. 2D). Follow-up analysis revealed no effect of group per each set size.

We next examined location memory by calculating the distance between the reported location of the item in the localization phase and the actual location of the item in the scene during the learning phase. Trials in which the objects were not correctly identified were excluded from localization analysis. There was no main effect of group [F(2,63) = 0.8, *p* = 0.45], but there was a significant interaction between set size and group [F(2,63) = 4.2, *p* = 0.02, η^2^_p_ = 0.12], as well as a significant main effect of set size [F(1,63) = 108, *p* < 0.001, η^2^_p_ = 0.6]. A follow-up analysis revealed a significant effect of group in set size 2 [F(2,63) = 3.16, *p* = 0.049, η^2^_p_ = 0.09, Fig. 2E] but not in set size 1 [F(2,63) = 0.02, *p* = 0.9].

## 4 Discussion

In the present study, we explored the effect of different APOE genotypes on both STM and LTM performance using sensitive tasks that have previously been successful in identifying subtle variations in performance in normal aging, neurodegenerative disorders, and at-risk cohorts (Rolinski et al., 2016, Salvato et al., 2016a, Salvato et al., 2016b, Zokaei et al., 2017). The results show impaired LTM for object locations in *ε4/ε4* carriers as indexed by the magnitude of placement error in the more demanding, 2-item condition (Fig. 2D). Paradoxically, STM for object locations, as measured by our task, was significantly better in *ε4/ε4* carriers (Fig. 1B). Considering the dissociable effects of the APOE gene on STM and LTM, it may be hypothesized that the 2 types of memories and the brain regions supporting these functions might not be co-extensive.

Impaired LTM performance in the current study in *ε4* carriers is in line with previous research demonstrating similar direction of findings both in healthy and AD patient carriers of the *ε4 as well as rodent studies on the effect of the APOE gene on spatial learning and memory* (Bour et al., 2008, Rodriguez et al., 2013). In patients with AD, *ε4* carriers display poorer LTM performance (Vlies et al., 2007, Wolk et al., 2010) as well as smaller hippocampal or other MTL regions volume (Geroldi et al., 1999, Hashimoto et al., 2001, Juottonen et al., 1998) even when groups are well matched for age and disease severity (Wolk et al., 2010). In the healthy older population, neuropsychological measures have yielded similar findings (Caselli et al., 2009, Caselli et al., 2011, Chey et al., 2000, Deary et al., 2002, Levy et al., 2004, Schultz et al., 2008), although not always replicable (Houston et al., 2005, Luciano et al., 2009, Wetter et al., 2005). Studies looking at object recognition and spatial navigation tests have yielded similar results, with noncarriers outperforming carriers in both task types (Berteau-Pavy et al., 2007, Haley et al., 2010). However, neuroimaging studies reporting a Blood-oxygen-level dependent signal reduction in hippocampal areas in carriers often fail to find any accompanying behavioral differences (Filippini et al., 2012, Gutchess et al., 2005).

Considering present findings, in the healthy population, it may be necessary to use sensitive and challenging tasks of LTM to identify the pattern of cognitive decline associated with the APOE gene rather than neuropsychological measures often used to identify deficits in disease rather than normal variations in health. LTM deficits, indexed by lower precision of memory for location and slower response times, were only observed in more difficult trials, that is, set size of 2.

Superior STM for object locations in our task is also supported by previous research (Carrión-Baralt et al., 2009, Greenwood et al., 2005, Greenwood et al., 2014, Wolk et al., 2010, Zokaei et al., 2017). In a study similar to the current investigation, middle-aged *ε4* carriers performed better than noncarriers (Zokaei et al., 2017). The interpretation of such an advantageous effect of *ε4* allele has not yet been resolved but one possibility is that it might constitute a compensatory mechanism. In other words, better STM performance may arise because of increased recruitment of regions that are not directly linked to prodromal stages of AD pathology, for example, frontal and parietal regions implicated in STM (Bookheimer et al., 2000). In fact, several studies have reported increased brain activity in these regions in *ε4* carriers when they performed STM tasks (Chen et al., 2013, Scheller et al., 2017, Wishart et al., 2006). According to such an explanation, with increasing age, structural and functional changes in brain areas subserving LTM processes (hippocampal and MTL regions) may lead to functional changes in brain regions supporting STM leading to enhanced performance in *ε4* carriers in these tasks. Behavioral data presented here may, to some extent, reflect such a compensatory mechanism.

Considering the dissociable effects of the APOE gene on STM and LTM, it may be hypothesized that the two types of memories and the brain regions supporting these functions might not be co-extensive. One recent model of STM proposes that this cognitive function can be perceived as an activated portion of the LTM (Cowan, 1998) and hence assume similar neural underpinnings for the two cognitive processes. This activated LTM state is in contrast to an item that is currently within the “focus of attention” and theoretically has distinguishable neural and behavioral mechanisms (Cowan, 2001). In line with such a proposal, the MTL has been reported to be active during the maintenance of more than one item in STM and in cases where fine-grained binding of features is required across different timescales (Axmacher et al., 2007, Libby et al., 2014, Oztekin et al., 2010, Yonelinas, 2013). Deficits after MTL damage have also been reported to result in impairments in both STM and LTM (Olson et al., 2006, Pertzov et al., 2013). The dissociable effects of the APOE gene on these 2 memory processes, however, highlight that these two mechanisms and their neural underpinnings are not fully co-extensive.

Enhanced STM performance, measured using the object-location task in the present study and the possible neuronal underpinnings of this effect may perhaps be mediated through other, not yet well-understood mechanisms associated with the APOE gene. In fact, several studies in a younger population have demonstrated enhanced cognitive performance in *ε*4 carriers relative to noncarriers in tasks of executive function, highlighting a more prominent dissociation between carriers and noncarriers beyond the context of AD and AD-related pathology (Marchant et al., 2010, Shaw et al., 2007). Furthermore, these phenotypical effects of the APOE gene can be explained within the antagonistic pleiotropy hypothesis. According to this hypothesis, the APOE *ε4* carriers might actually be at an advantage earlier in life, with detrimental traits associated with the allele evident only at a point beyond normal reproductive age. It is important to note that the evidence for the mechanisms underlying this is not yet fully known, although a few studies in rodent models of APOE are in line with this hypothesis (Moreau et al., 2013).

Moreover, it is important to note that the present study does not examine the effect of another APOE gene allele, the *ε*2 allele, on memory performance. In contrast to the *ε*4 allele, the *ε*2 allele APOE gene is protective against AD (see Suri et al., 2013, for a review of evidence). Recently, it has been shown that younger carriers of *ε*2 allele have more gray matter in the hippocampus and rely more on hippocampal-dependent strategies in tasks of navigation than noncarriers (Konishi et al., 2016). In contrast, other studies have found performance disadvantages in attention-related tasks in *ε*2 carriers in middle and old age, compared with *ε*3 carriers (Lancaster et al., 2016, Lancaster et al., 2017). Considering these complicated effects of the APOE *ε*2 allele on cognitive performance, future studies should aim to examine the role of all APOE alleles on behavior, across aging, to provide a more comprehensive phenotypical description of this gene on behavior and its relationship with AD.

Together, our findings provide support for both beneficial and detrimental effects of the APOE ε4 gene allele on cognition in healthy older participants. This was possible due to the tasks used to measure both STM and LTM, which used a continuous, analogue report and were sensitive to subtle variations in performance. Importantly, although the detrimental effects on LTM performance may be explained in terms of AD-related pathology, the superior STM performance can either highlight a compensatory mechanism or in turn hint to independent, phenotypical effects of this allele on cognition. Further studies should first aim to replicate these findings in other age groups (both younger and older participants) and expand on these findings by similarly examining relationships between various cognitive functions across the life span using sensitive, cutting-edge behavioral measures and ideally using designs that equate stimulus properties and response demands.

## Disclosure statement

There are no actual or potential conflicts of interest.
